# Differentiation between Enamines and Tautomerizable Imines Oxidation Reaction Mechanism using Electron-Vibration-Vibration Two Dimensional Infrared Spectroscopy

**DOI:** 10.3390/molecules24050869

**Published:** 2019-03-01

**Authors:** Fengqin Long, Zheng Chen, Keli Han, Lu Zhang, Wei Zhuang

**Affiliations:** 1 State Key Laboratory of Molecular Reaction Dynamics, Dalian Institute of Chemical Physics, Chinese Academy of Sciences, Dalian 116023, China; fqlong@dicp.ac.cn; 2 University of the Chinese Academy of Sciences, Beijing 100049, China; 3 State Key Laboratory of Structural Chemistry, Fujian Institute of Research on the Structure of Matter, Chinese Academy of Sciences, Fuzhou 350002, China; jeack1808@126.com; 4 Collaborative Innovation Center of Chemistry for Energy Materials, Shanghai Key Laboratory of Molecular Catalysis and Innovative Materials, MOE Key Laboratory of Computational Physical Sciences, Department of Chemistry, Fudan University, Shanghai 200433, China

**Keywords:** electron-vibration-vibration two dimensional spectroscopy, reaction intermediate, enamine, imine

## Abstract

Intermediates lie at the center of chemical reaction mechanisms. However, detecting intermediates in an organic reaction and understanding its role in reaction mechanisms remains a big challenge. In this paper, we used the theoretical calculations to explore the potential of the electron-vibration-vibration two-dimensional infrared (EVV-2DIR) spectroscopy in detecting the intermediates in the oxidation reactions of enamines and tautomerizable imines with 2,2,6,6-tetramethylpiperidine-1-oxyl (TEMPO). We show that while it is difficult to identify the intermediates from their infrared and Raman signals, the simulated EVV-2DIR spectra of these intermediates have well resolved spectral features, which are absent in the signals of reactants and products. These characteristic spectral signatures can, therefore, be used to reveal the reaction mechanism as well as monitor the reaction progress. Our work suggests the potential strength of EVV-2DIR technique in studying the molecular mechanism of organic reactions in general.

## 1. Introduction

One of the great challenges in chemistry is to identify the structure and behavior of intermediates in the multi-step reactions. Since the intermediates are highly reactive and have short lifetimes, it is difficult, in general, to isolate and investigate them. The techniques commonly employed for this purpose include UV/Vis [1,2], infrared (IR) [3,4,5,6,7], electron paramagnetic resonance (EPR) [8,9,10,11] as well as nuclear magnetic resonance (NMR) spectroscopy [12,13]. Among these techniques, infrared (IR) spectroscopy is one of the most convenient tools. It has been successfully used, for instance, to detect the intermediate species formed in the water-oxidation mechanism on a haematite surface [14]. In addition, the in situ IR spectrum was used to detect the key intermediate Et_3_SiK in the reaction of KOt-Bu and Et_3_SiH in THF [15]. However, vibration modes of complex molecular structures usually lead to sophisticated features in the one-dimensional IR spectra that jam together and prevent the peak assignment. 

Over the past decade, coherent multidimensional vibrational spectroscopy has been developed into a powerful tool in understanding the solution chemistry and physics at the molecular level. These multidimensional analogues of their NMR counterparts can trigger various photophysical and photochemical processes, such as hydrogen bond dynamics in water [16,17,18], conformational transformation [19,20] and chemical exchange [21,22,23], which are interesting on their own. Multidimensional vibrational spectroscopy measures the signals arise from vibrational couplings between multiple quantum coherences excited by two or more tunable excitation pluses. These techniques can excite any vibrational combination bands or electronic states through the coherence pathways. When one excitation perturbs the other, a cross peak occurs between the states. This spreading of spectroscopic features into two dimensions reduces, or in many cases eliminates, the congestion in the signals [24,25].

The electron-vibration-vibration two-dimensional infrared (EVV-2DIR) spectroscopy is one of the frequency domain two-dimensional vibrational techniques. EVV-2DIR is a hybrid IR-Raman method which use two picosecond infrared pulses and one picosecond visible pulse to induce a third–order nonlinear optical response on a sample with frequency components ω =|±ω_α_±ω_β_±ω_γ_|. When the two variable IR pluses frequencies are resonant with coupled vibration modes, the nonlinear polarization can radiate a multiplicatively enhanced signal from the sample with frequency ω [25,26,27]. In other words, the final signal intensities not only rely on the vibrational states of the molecules and but also rely on their electronic states. EVV-2DIR spectroscopy considerably enhanced spectral decongestion since it can spread information in two dimensions and probe only coupled vibrational modes. These characteristics make it a useful tool for studying complex mixtures or complex structures [28,29]. 

In this paper, we demonstrate, by theoretical calculation, that EVV-2DIR can help to detect the intermediates in the oxidation reaction of enamines and tautomerizable imines with 2,2,6,6-tetramethylpiperidine-1-oxyl (TEMPO). Imine and enamine are two of the most common reaction intermediates in organic reactions [30]. A well-accepted picture is that the imine intermediates containing α-hydrogen generally exhibit similar reactivity to enamines since they can tautomerize to their enamine tautomers [31,32,33]. In a recent work, however, we demonstrated that the chemical and region-selectivity of the enamine tautomer derived from tautomerizable imine and the real enamine are different in the TEMPO reaction [34]. This difference was suggested, mainly by DFT calculation, to be due to the flexible imine-enamine tautomerization of the imine intermediate containing α-hydrogen. The experimental characterization of these reaction pathways and the existence of the important intermediates, similar as in most of the organic chemical reaction studies, are highly non-trivial and pleading for novel techniques.

Based on the previously calculated reaction mechanism, we calculated the IR, Raman and EVV-2DIR spectra of reactants, intermediates, products on the reaction pathway of imine and enamine with TEMPO by ab initio calculations. Assignment of the spectral peaks for intermediates in the conventional IR and Raman spectra is found to be difficult, while all of the intermediates exhibit well resolved, non-overlapping characteristic cross peaks in the EVV-2DIR spectrum. We proposed that these spectral features can be used to reveal the reaction pathways and monitor the reaction progress. Further analysis leads to the structural origins of these cross peaks. Our work, therefore, proposes that EVV-2DIR is a potentially powerful means to identify the reaction mechanism of complex organic reactions.

## 2. Results

### 2.1. Calculating Vibrational Spectra of Key Species along the Pathway of Enamine Oxidation Reaction

**Figure 1:**
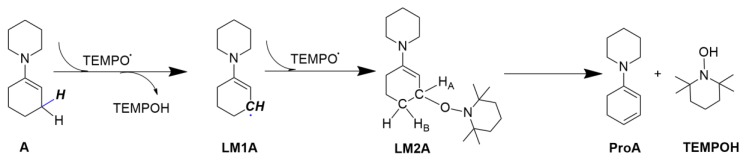
Calculated elementary steps for oxidation of enamine molecule A by TEMPO.

**Figure 2:**
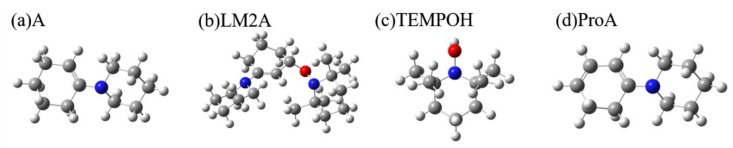
Calculated geometries of (**a**) reactant A, (**b**) β-aminoxylated intermediate LM2A, (**c**) TEMPOH and (**d**) amino diene intermediate ProA.

Figure 1 presents the calculated elementary steps for the oxidation of enamine by TEMPO. We first calculated the IR and Raman spectra of all the reaction-involved species and examined whether there are significant features that can differentiate the β-aminoxylated intermediate LM2A from the reactant enamine molecule A, TEMPOH product, and amino diene intermediate ProA that is a precursor to aryl amine product in the reaction pathway. Herein, we do not show the spectra of reactant TEMPO and intermediate LM1A in here, since for both of them are free radical and the calculated maximum signal intensity are two orders of magnitude smaller than that of ProA in 2DIR spectra. The calculated geometries of each species are shown in Figure 2.

#### 2.1.1. Calculated IR and Raman Spectra

**Figure 3:**
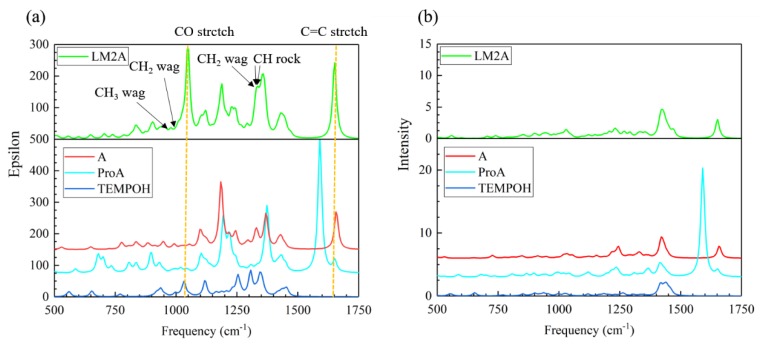
Calculated (**a**) IR and (**b**) Raman spectrum for each species in the enamine reaction pathway. The half-width at half height of IR and Raman peaks is 10 cm^−1^.

In the IR and Raman signals of these four species, most of the vibration modes appear in two regions, 500–1750 cm^−1^ and 2750–3250 cm^−1^ (see Supporting Information Figure S1). The peaks between 2750–3250 cm^−1^, originate from the C-H symmetric and antisymmetric stretching vibration of the six-membered ring, are similar for all these species. For the regime between 500–1750 cm^−1^ (Figure 3), two strong absorption peaks at 1050 and 1650 cm^−1^ are observed in the IR signals of the β-aminoxylated intermediate LM2A. The vibration mode at 1050 cm^−1^, originated from the C-O stretch vibration, does not exist in the enamine A and amino diene intermediate ProA. However, this peak is also contributed by the N-O stretching of TEMPOH. Hence, assignment of the spectral features and using it as a probe of LM2A becomes inconclusive. The peak at 1650 cm^−1^, related to C=C stretch vibration, also overlaps with the C=C stretching of enamine molecule A and amino diene species ProA. Other strong peaks of LM2A within 1000 cm^−1^ to 1750 cm^−1^, also overlap with the peaks in other species. It is therefore difficult to identify specific spectra feature for LM2A in the conventional IR spectra. A similar situation is observed in the Raman spectroscopy in which all peaks of the intermediate LM2A overlap with those of others.

#### 2.1.2. Calculated EVV-2DIR Spectrum in Enamine Reaction Pathway

**Figure 4:**
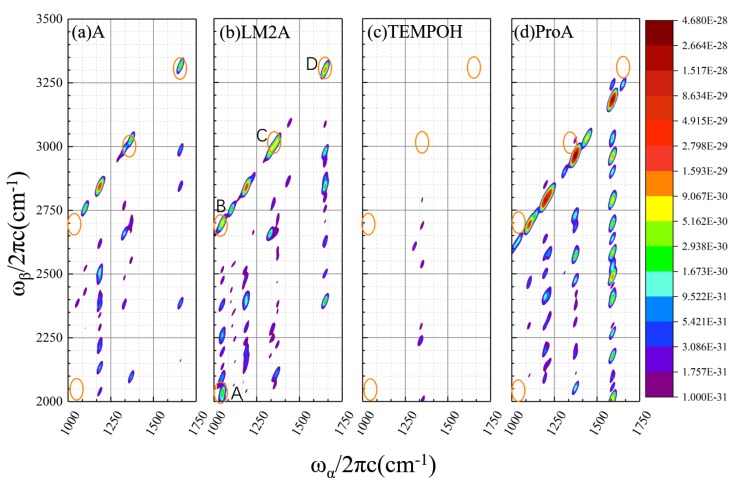
Calculated EVV-2DIR spectrum for (**a**) reactant A, (**b**) β-aminoxylated intermediate LM2A, (**c**) TEMPOH and (**d**) amino diene intermediate ProA in enamine reaction pathway. The figure is shown as a contour map with a logarithmic scale. The characteristic cross peaks in the intermediate LM2A are indicated by labels and orange circles.

We next examined the spectral features, in the EVV-2DIR spectra, between 1000–1750 cm^-1^ (Figure 4). The simulated EVV-2DIR spectra of the key species in enamine reaction pathway, including the reactant enamine molecule A, β-aminoxylated intermediates LM2A, amino diene intermediate ProA and TEMPOH in the enamine reaction pathway are shown in Figure 4. The ω_α_/2πc and ω_β_/2πc correspond to the fundamental vibrational mode and combinational band (or overtone band), respectively. And the Z-axis shows the absolute intensity of the cross peak for a single molecule. There are four prominent cross peaks only present in the spectra of intermediate LM2A, labeled as A to D (A and B located at ω_α_ = 1050 cm^−1^, ω_β_ = 2035 and 2700 cm^−1^, respectively. C and D located at 1335/2985 cm^−1^ and 1645/3295 cm^−1^). Note that we define the characteristic cross peak as that shows intensity at least an order of magnitude higher than other species.

To reveal the underlying molecular nature of each characteristic peak, we calculated the contribution ratio of each coupling modes to the signal intensity of characteristic peak (methods described in Section 4.1), and assign the vibration modes. For peak A, two coupling modes 1050.2/2030.7 cm^−1^ and 1050.2/2047.8 cm^−1^ contribute 81% and 17% to the total signal intensity, respectively (Table 1). For the peaks B and D, only one pair of vibration mode contributes dominantly (more than 96%) to the signal intensity. Three pairs contribute significantly (each about 30%) to the intensity of peak C. Peaks A and B located along the vertical axis at ω_α_ = 1050.2 cm^−1^. The peak A originate from the coupling of ω_α_ = 1050.2 cm^−1^ and ω_β_ = 2030.7 and 2047.8 cm^−1^, which can be assigned as the infrared active asymmetry C-O stretch mode and combination bands of C-O stretching with the Raman active CH_2_ wag mode (for ω_β_ = 2030.7 cm^−1^) and C-O stretching with the CH_3_ wag mode (for ω_β_ = 2047.8 cm^−1^). Peak B involves the fundamental C-O stretch mode with the combination band of C-O stretching and C=C stretching. Peak C mainly originates from the CH rock and binary combination bands of CH rock + C=C stretch and CH_2_ wag + C=C stretch. The C=C stretch fundamental vibration mode at 1650.1 cm^−1^ coupled with its first overtone (3300.2 cm^−1^) contributes to the signal intensity of peak D.

In the EVV-2DIR spectra, excitation of the combination band owing to two types of anharmonic coupling: mechanical anharmonic coupling of the IR and Raman active vibration modes and electric anharmonic coupling which is the nonlinear dependence of the molecular dipole moment with respect to the vibrational coordinates. For peaks A, C and D, the contributions from χ^mech^ and χ^elect^ are comparable (Table 1). Therefore, we should consider both mechanisms to determine the total susceptibility. For peak B, the electric anharmonicity is dominant, therefore its features are mainly related to the coupling between the CO stretch mode and C=C stretch mode. 

It is important to note that the cross peaks in EVV-2DIR only probe coupled vibrational modes. Though there is a peak at ~1050 cm^−1^ in the IR spectra of TEMPOH, no cross peaks appear at a corresponding position in the 2DIR spectra. The absence of these cross peaks is due to the lack of Raman active modes at 980.5, 997.6 and 1650.0 cm^−1^ couple with the N-O stretching mode in TEMPOH. This is a clear demonstration of the ability of EVV-2DIR spectra enhance spectral decongestion. The cross peaks A and B are strong evidence for the existence of intermediate LM2A. 

### 2.2. Calculating Vibrational Spectra of Key Species along the Pathway of Imine Oxidation Reaction

**Figure 5:**
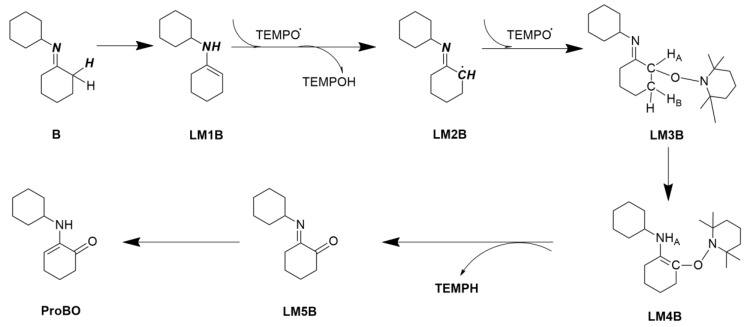
Calculated elementary steps for oxidation of imine molecule B by TEMPO.

**Figure 6:**
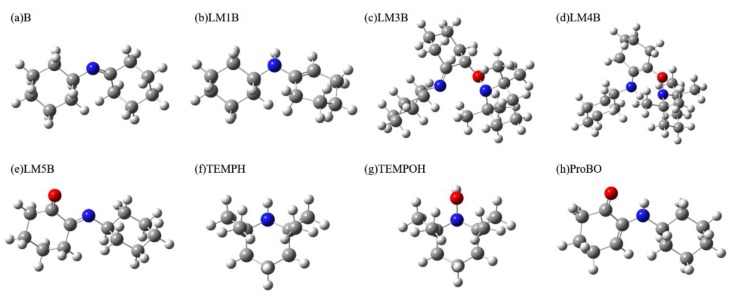
Calculated geometries of (**a**) reactant imine molecule B, (**b**) enamine tautomer intermediate LM1B, (**c**) α-aminoxylated species intermediate LM3B, (**d**) α-aminoxylated enamine intermediate LM4B, (**e**) intermediate LM5B, (**f**) TEMPH, (**g**) TEMPOH and (**h**) α-amino-enones product ProBO.

Figure 5 shows the calculated elementary steps for oxidation of imine by TEMPO. Identifying the spectral features of the key intermediates enamine tautomer LM1B, α-aminoxylated species LM3B and α-aminoxylated enamine species LM4B can help infer the reaction mechanism. Again, the IR and Raman spectrum of reactant imine molecule B, intermediates LM1B, M2B to LM5B, TEMPH, TEMPOH, and product α-amino-enones ProBO were calculated (The spectra of free radical TEMPO and LM2B will be difficult to detect in the experiment, therefore they are not considered). The calculated geometries of each species are shown in Figure 6. 

#### 2.2.1. Calculated IR and Raman Spectrum in the Imine Reaction Pathway

**Figure 7:**
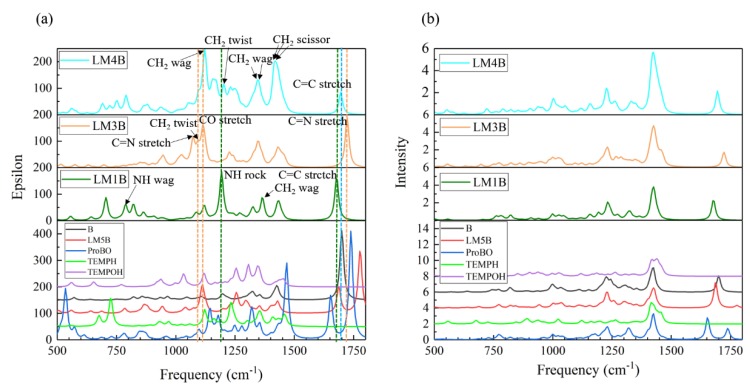
Calculated IR (**a**) and Raman (**b**) spectrum for each species in imine reaction pathway. The half-width at half height of IR and Raman peaks is 10 cm^−1^.

Figure 7 presents the calculated IR and Raman spectra for the aforementioned eight key species in the imine reaction pathway. The peaks in the range of 2750–3250 cm^−1^ (Supporting Information Figure S2), arise from the C-H symmetric and antisymmetric stretching vibration of the six-membered ring in each species, almost completely overlap in these species. We, therefore, examined the vibration modes in the region of 500–1800 cm^−1^. 

There are two strong peaks at 1192 and 1677 cm^−1^ in the IR spectra of the enamine species LM1B, which can be assigned to NH rock and C=C stretching mode, respectively. CH_2_ twist of the α-aminoxylated enamine species LM4B also generates a peak at ~1192 cm^−1^. The peak at 1677 cm^−1^, which does not appear in other spectra, can be used to identify LM1B. However, the CH_2_ twist vibration mode of intermediate LM5B is adjacent to it, which interferes with spectral identification. Other peaks of LM1B within 1000 cm^−1^ to 1800 cm^−1^ also overlap with the peaks from other species. In the IR spectra of the α-aminoxylated intermediate LM3B, the CO stretch and C=N stretch contribute to two strong peaks at 1074/1091 and 1723 cm^−1^. However, the CH_2_ wag of LM4B also contribute a comparable strong peak at ~1074 cm^−1^ and this peak also overlaps with the CH_2_ twist of LM1B, LM5B, TEMPOH and TEMPH. The peak at ~1723 cm^−1^ does not overlap with other species, but the adjacent vibrational mode of C=N stretch of imine B and C=O stretch of α-amino-enones ProBO make the spectra assignment become elusive. In the IR spectra of LM4B, four strong absorption peaks at 1124, 1342, 1419 and 1695 cm^−1^ are observed. However, almost all peaks including these four peaks overlap with the peaks in other species. 

Therefore, it is hard to identify the specific spectral features for each intermediate from the IR signal. The spectra congestion is more serious in the Raman spectra. 

#### 2.2.2. Calculated EVV-2DIR Spectrum in Imine Reaction Pathway

**Figure 8:**
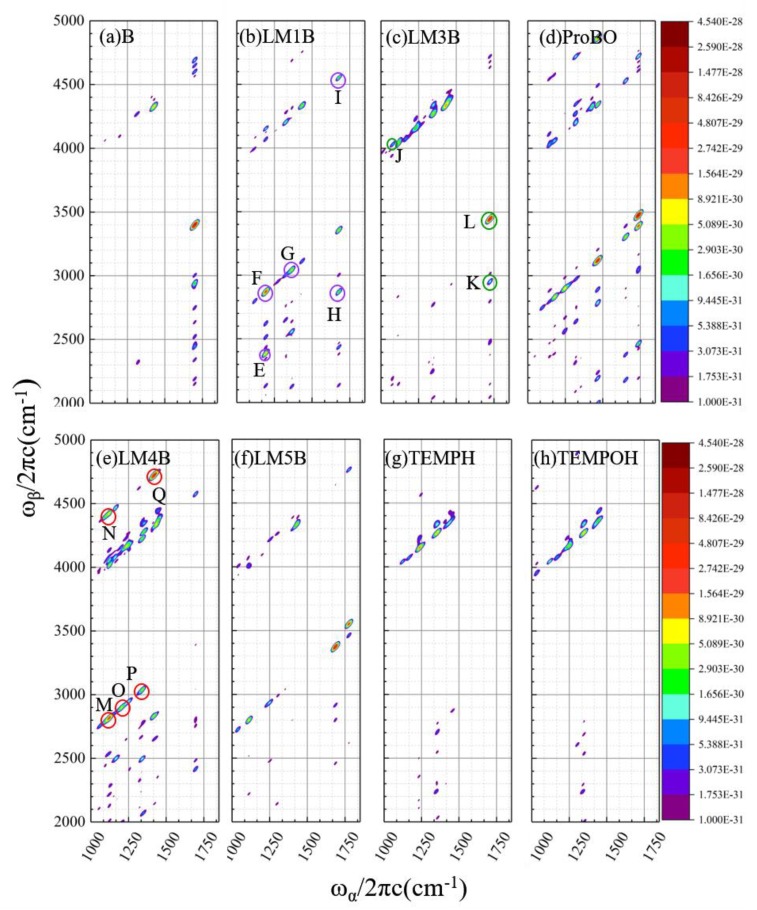
Calculated EVV-2DIR spectrum for (**a**) reactant imine molecule B, (**b**) enamine tautomer intermediate LM1B, (**c**) α-aminoxylated species intermediate LM3B, (**d**) α-amino-enones product ProBO, (**e**) α-aminoxylated enamine intermediate LM4B, (**f**) intermediate LM5B, (**g**) TEMPH and (**h**) TEMPOH in tautomerizable imine reaction pathway. The figure is shown as a contour map with a logarithmic scale. The characteristic cross peaks in the intermediate LM1B are indicated by labels and purple circles, in the α-aminoxylated intermediate LM3B are indicated by labels and green circles and in the α-aminoxylated intermediate LM4B are indicated by labels and red circles.

Shown in Figure 8 are the calculated EVV-2DIR spectra for each species in tautomerizable imine reaction pathway, in a window from 1000 cm^−1^ to 1800 cm^−1^. We next identified the feature peaks only presented in the signals of enamine species LM1B, α-aminoxylated intermediate LM3B and LM4B, respectively. In addition, we calculated the contribution ratio of each vibration mode pairs to the signal intensity of characteristic peak (methods described in Section 4.1), and assigned the vibration mode pairs. In addition, we also calculated the mechanical anharmonicity χ^mech^ and electrical anharmonicity χ^elect^ contributions of each coupling modes to the cross peak intensity (shown in Table 2, Table 3 and Table 4).

#### 2.2.3. Analysis of the Characteristic Cross Peaks of Intermediates in Oxidation Reaction of Imine

Five feature cross peaks are identified in the enamine tautomer LM1B (Figure 8b), labeled as E to I. All of them are composed of one vibration mode pair which makes more than 96% contribution to the intensity of the corresponding cross peak. The peak E is contributed by the NH rock vibration mode at 1192.6 cm^−1^ as well as a first overtone (2385.2 cm^−1^). The peak F originates from NH rock vibration mode (1192.6 cm^−1^), together with the combination band (2870.4 cm^−1^) from NH rock and C=C stretch. The peaks G arises from the CH_2_ wag fundamental at 1365.5 cm^−1^ and combination band at 3043.3 cm^−1^, which can be assigned to CH_2_ wag + C=C stretch. Another two peaks (H and I) arise from the C=C stretch fundamental at 1677.8 cm^−1^ and combination band at 2870.4 cm^−1^ and 4556.6 cm^−1^, which can be assigned to C=C stretch + NH rock and CH_2_ symmetric stretch + C=C stretch, respectively. 

Though there is a cross peak at 1162/2870 cm^−1^ in the α-aminoxylated enamine species LM4B, which is in the same position as the peak F in LM1B, the signal intensity of the cross peak in LM1B are defined at least one order of magnitude higher than that in LM4B. And no cross peak appears at the corresponding position of peak E in LM4B, because LM4B do not have Raman active mode at ~1193 cm^−1^. The peak I in LM1B also appears in the corresponding position in ProBO and LM4B, while the intensity in LM1B is much higher than that of the other two species. The peaks G and H in LM1B also show very weak intensities at the corresponding positions in ProBO and LM5B.

For peaks F, H and I, the susceptibility only determined by the through-space-induced electrical anharmonic coupling and the mechanical anharmonicity is negligible compared with that from the electric anharmonicity. The susceptibility from two types of anharmonicity are quantitatively similar for peaks E and G, so both kinds of anharmonicity contribute to the signal intensity.

Three feature cross peaks of α-aminoxylated species LM3B are shown in Figure 8c, labeled as J to L. Five coupling vibration modes contribute to peak J, and the contribution ratio ranges from 8% to 30%. The five vibration mode pairs’ contributions can be classified as two vibrational assignments: one is C=N stretch mode at 1074.4 cm^−1^ and combination band assigned to C=N stretch + CH_2_ asymmtric stretch; the other is the mode of CH_2_ twist at 1091.8 cm^−1^, as well as the combination band involving CH_2_ twist +CH_2_ asymmetric stretch. There is only one vibration mode pair contributing to either peaks K or L. The cross peak K arises from the C=N stretch fundamental at 1723.5 cm^−1^ and the combination bands (2954.3cm^−1^) of C=N stretch + CH_2_ twist. The peak C (1723.5/3446.9 cm^−1^) arises from the C=N stretch fundamental and its first overtone. 

The cross peak J is only present in LM3B. Though there is a peak at ~1074/1091 cm^−1^ in IR spectra of LM1B, LM4B, LM5B, TEMPOH and TEMPH, the absence of the cross peak in EVV-2DIR spectra in these species indicate that they don’t have Raman active vibration mode at 2962, 2951 and 2947 cm^−1^. The peak K in LM3B shows very weak intensity at the corresponding positions in reactant imine B. The peak L in LM3B is strong, but imine B and α-amino-enones ProBO both have peaks with comparable intensity in the adjacent positions. Therefore, the peak L is difficult to distinguish from other species, and peaks J and K are characteristics for LM3B.

The peak J mainly arise from the nonlinearity of the molecular dipole moment along vibrational coordinates. The two distinctive contributions χ^mech^ and χ^elect^ are comparable for peaks K and L.

Figure 8e presents five key cross peaks in the α-aminoxylated enamine species LM4B, labeled as M to Q. The peaks M and N both arise from the CH_2_ wag fundamental at 1124.51 cm^−1^ coupled with combination bands 2820.03 cm^−1^ and 4427.14 cm^−1^, respectively; the peak O originates from the CH_2_ twist mode at 1203.0 cm^−1^ and combination band (2898.5cm^−1^) from CH_2_ twist + C=C stretch. The peak P is contributed by two vibration mode pairs, both arise from the CH_2_ wag and combination band CH_2_ wag + C==C stretch. The peak Q arises from three vibration mode pairs, all of which are composed of CH_2_ scissor and combination band from CH_2_ scissor +NH stretch.

Though almost all peaks in LM4B overlap with the peaks in other species in the conventional IR spectra, the cross peaks N and Q are only present in LM4B in 2DIR spectra. The cross peaks M, O and P in LM4B also appear in the corresponding position for LM1B and LM5B, while with very weak intensity. Identifying the cross peaks N and Q can provide strong evidence of the existence of intermediate LM4B. 

The electric anharmonicity is dominant for all the five cross peaks, which imply that the interactions of the coupling modes are strong for all of them.

## 3. Discussion

In the case of the oxidation reaction of enamine with TEMPO, the EVV-2DIR spectra in the range of 1000~1750 cm^−1^ is relatively sparseness compared with the one dimension spectra within the same region. This is due to the fact that the EVV-2DIR spectroscopy involved the direct excitation of vibrational combination bands which stem from the anharmonic coupling of two vibrational modes. For instance, only the coherence decay involving the CO stretch mode at 1050.2 cm^−1^ is detected for the β-aminoxylated intermediate LM2A, while the other vibration modes are filtered out because they are not interacting with the second pulse ω_β_. The CO stretching mode contributes a strong absorption peak at ~1050.2 cm^−1^ in the IR spectra of LM2A and this vibration mode does not exist in enamine molecule A and amino diene species ProA. However, this peak is also contributed by the NO stretching of TEMPOH, inhibiting its application as a probe of LM2A. However, in the EVV-2DIR, there are two significant feature peaks involving the CO stretch as the center frequency (peaks A and B in Figure 4), which do not present in the spectra of any other species. Therefore, the cross peaks A and B can be evidence of the existence of intermediate LM2A. Our calculations predict that the EVV experiment can measure the ω_α_ ~ 1050 cm^−1^ to detect the intermediate LM2A.

The oxidation reaction of tautomerizable imine with TEMPO involve six elementary reactions and ten species in the reaction pathway. The IR spectra of enamine tautomer intermediates LM1B, α-aminoxylated intermediate LM3B and α-aminoxylated enamine intermediate LM4B display extremely similar absorption peaks. However, the situation in the two-dimensional EVV spectra has been greatly improved. Four distinct characteristic peaks are presented for LM1B (Figure 8b), which arise from either the NH wag coupled with the combination band NH wag + C=C stretch or the NH rock involved with the first overtone; three feature peaks are displayed for LM3B (Figure 8c), which arise from the CO stretch and the combination bands CO stretch + CH_3_ wag, as well as C=N stretch involved combination band C=N stretch and CH_2_ asymmetric stretch; five feature peaks are shown for LM4B (Figure 8e), which originate from the CH_2_ wag (twist or scissor) together with the combination bands from CH_2_ wag (twist or scissor) + C=C stretch or CH_2_ wag (twist or scissor) + NH stretch. It is clear to see that all the feature peaks presented in Figure 8b,c,e arise from different vibration modes, so the intermediates LM1B, LM3B and LM4B can be identified and assigned easily. 

As shown above, the EVV-2DIR spectrum can serve as a useful tool to identify the intermediates in the oxidation reaction of enamine/tautomerizable imine with TEMPO. Furthermore, identification of the intermediates using the EVV-2DIR spectrum clearly demonstrates the reactions of enamine and tautomerizable imine with TEMPO follow different reaction pathways. The results altogether suggest the EVV-2DIR spectrum holds great potential in investigating the reaction in complex mixtures.

## 4. Materials and Methods 

### 4.1. EVV-2DIR Spectroscopy

EVV-2DIR spectroscopy, also known as doubly vibrational enhanced four-wave mixing (DOVE-FWM), is a coherent multidimensional spectroscopy first reported by Wright and co-workers [26,27,28,35] and has been applied to a range of biological and chemical problem including proteomics [36], imaging and structural analysis [37,38]. 

**Figure 9:**
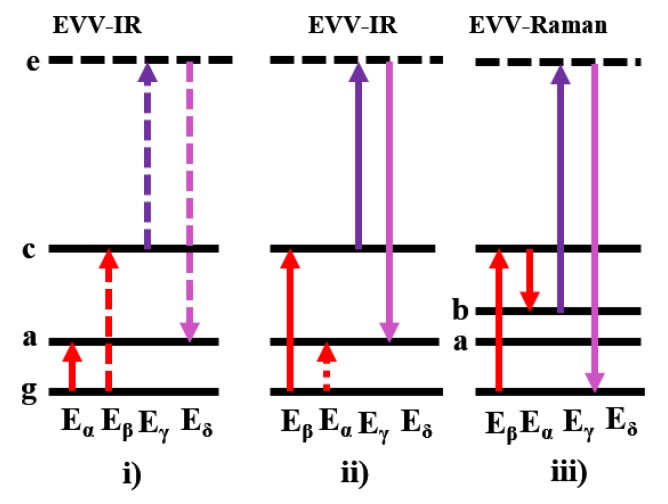
Three coherent pathways contributing to the EVV-2DIR signal, labeled as **i**)–**iii**). Here, g denotes ground state, a and b denote vibrational states, c denotes their combined state and e denotes virtual electronic state. ω_α_ and ω_β_ are always pulses 1 or 2 and defined such that ω_β_ > ω_α_. The pathway (**i**) is for ω_α_ arrive at the sample first, and the pathways 2 and 3 are for ω_β_ arriving first. Solid arrows show ket transition and dotted arrows show bra transitions.

EVV-2DIR spectroscopy uses three independently tunable picosecond pulsed laser beams to induce a third–order nonlinear optical response on a sample in a phase-matched condition. Two of the pulses are infrared, which excite molecular vibrations, and the third one is a visible beam that is used to detect the polarization induced by the two infrared beams. When the frequencies of the two infrared beams are scanned independently, if the two IR pulses are in resonance with coupled vibrational states, the detected signal will be significantly enhanced. Therefore, in this regime, both vibrational polarization and electronics contribute to the third–order nonlinear susceptibility χ^(3)^. There are three coherence pathways contributing to the total EVV-2DIR signal intensity (Figure 9). We can choose the pathway (i) by use the infrared pulse ω_α_ as the first one to excite the molecule ground state.

Here, we focus on the EVV-2DIR coherence pathway (i) and the excitation fields are defined as:

(1) $$E_{i}=\frac{1}{2}\left[E_{i}^{0}e^{i(k_{i}\cdot r-\omega_{i}t)}+E_{i}^{0*}e^{-i(k_{i}\cdot r-\omega_{i}t)}\right]$$

where the $E_{i}^{0}$ denotes the amplitudes.

Using the time-dependent perturbation theory [39], the corresponding third-order density operator can be written as:

(2) $$\begin{array}{ll} \rho_{\left(i\right)}^{\left(3\right)}(t)= & -\frac{1}{8}{\left(\frac{1}{\hbar }\right)}^{3}\frac{|e\rangle \langle e\left|\mu \right|c\rangle \langle c\left|\mu \right|g\rangle \langle g\left|\mu \right|a\rangle \langle a|e^{-i\omega_{s}t}E_{1}^{0}E_{2}^{0*}E_{3}^{0}}{\left(\omega_{\alpha }-\omega_{cg}+i\Gamma_{cg}\right)\left(\omega_{\alpha }-\omega_{\beta }-\omega_{ca}+i\Gamma_{ca}\right)\left(\omega_{\alpha }-\omega_{\beta }+\omega_{\gamma }-\omega_{ea}+i\Gamma_{ea}\right)}\\ & -\frac{1}{8}{\left(\frac{1}{\hbar }\right)}^{3}\frac{|e\rangle \langle e\left|\mu \right|c\rangle \langle c\left|\mu \right|g\rangle \langle g\left|\mu \right|a\rangle \langle a|e^{-i\omega_{s}t}E_{1}^{0}E_{2}^{0*}E_{3}^{0}}{\left(-\omega_{\beta }-\omega_{ga}+i\Gamma_{ga}\right)\left(\omega_{\alpha }-\omega_{\beta }-\omega_{ca}+i\Gamma_{ca}\right)\left(\omega_{\alpha }-\omega_{\beta }+\omega_{\gamma }-\omega_{ea}+i\Gamma_{ea}\right)}\\ & -\frac{1}{8}{\left(\frac{1}{\hbar }\right)}^{3}\frac{|e\rangle \langle e\left|\mu \right|c\rangle \langle c\left|\mu \right|g\rangle \langle g\left|\mu \right|a\rangle \langle a|e^{-i\omega_{s}t}E_{1}^{0}E_{2}^{0*}E_{3}^{0}}{\left(\omega_{\alpha }-\omega_{cg}+i\Gamma_{cg}\right)\left(\omega_{\alpha }-\omega_{\beta }-\omega_{ca}+i\Gamma_{ca}\right)\left(\omega_{\alpha }-\omega_{\beta }-\omega_{s}-\omega_{ea}+i\Gamma_{ea}\right)}\\ & -\frac{1}{8}{\left(\frac{1}{\hbar }\right)}^{3}\frac{|e\rangle \langle e\left|\mu \right|c\rangle \langle c\left|\mu \right|g\rangle \langle g\left|\mu \right|a\rangle \langle a|e^{-i\omega_{s}t}E_{1}^{0}E_{2}^{0*}E_{3}^{0}}{\left(-\omega_{2}-\omega_{ga}+i\Gamma_{ga}\right)\left(\omega_{1}-\omega_{2}-\omega_{ca}+i\Gamma_{ca}\right)\left(\omega_{1}-\omega_{2}-\omega_{s}-\omega_{ea}+i\Gamma_{ea}\right)} \end{array}$$

where ω_n_ is the frequency of pulse n, $\omega_{\mathrm{ij}}$ and $\Gamma_{\mathrm{ij}}$ are the frequency difference and the dephasing rate for the i-j transition, μ is dipole moment operator.

The material polarization can be written as:

(3) $$P=\langle \mu \rho^{\left(3\right)}(t)\rangle =\varepsilon_{0}\chi^{\left(3\right)}\cdot E_{\alpha }\cdot E_{\beta }\cdot E_{\gamma }$$

So, by invoking the Placzek approximation [40], we can obtain the third-order susceptibility:

(4) $$\begin{array}{ll} \chi_{\left(i\right)}^{\left(3\right)}= & -\frac{NF^{\left(3\right)}}{48}{\left(\frac{1}{\hbar }\right)}^{2}\frac{\langle a|\alpha |c\rangle \langle c|\mu |g\rangle \langle g|\mu |a\rangle }{\left(\omega_{1}-\omega_{2}-\omega_{ca}+i\Gamma_{ca}\right)\left(\omega_{1}-\omega_{cg}+i\Gamma_{cg}\right)}\\ & -\frac{NF^{\left(3\right)}}{48}{\left(\frac{1}{\hbar }\right)}^{2}\frac{\langle a|\alpha |c\rangle \langle c|\mu |g\rangle \langle g|\mu |a\rangle }{\left(\omega_{1}-\omega_{2}-\omega_{ca}+i\Gamma_{ca}\right)\left(-\omega_{2}-\omega_{ga}+i\Gamma_{ga}\right)} \end{array}$$

where the α is polarizability operator, N is number density of molecules and F^(3)^ is the local field correction.

Using the Taylor series to expand the dipole moment operator and polarizability operator with respect to vibrational coordinates Q of every mode, (Equations 5 and 6), and only the lowest-order contributions are considered, the susceptibility can be written as a sum of two distinctive terms:

(5) $$\mu =\mu_{0}+\sum_{a}{\left(\frac{\partial \mu }{\partial Q_{a}}\right)}_{0}Q_{a}+\frac{1}{2}\sum_{a,b}{\left(\frac{\partial^{2}\mu }{\partial Q_{a}\partial Q_{b}}\right)}_{0}Q_{a}Q_{b}+\ldots$$

(6) $$\alpha =\alpha_{0}+\sum_{a}{\left(\frac{\partial \alpha }{\partial Q_{a}}\right)}_{0}Q_{a}+\frac{1}{2}\sum_{a,b}{\left(\frac{\partial^{2}\alpha }{\partial Q_{a}\partial Q_{b}}\right)}_{0}Q_{a}Q_{b}+\ldots$$

(7) $$\chi_{\left(i\right)}^{\left(3\right)}≅\chi_{\left(i\right)}^{mech}+\chi_{\left(i\right)}^{elect}$$

The first part is mechanical anharmonicity which arises from the lowest-order anharmonic terms in molecular potential energy surface, while the second part is known as electricity anharmonicity, arising from the nonlinearity of the dipole moment with respect to the vibrational coordinates, known as electrical anharmonicity. The mechanical anharmonicity contribution and the electrical anharmonicity contribution can be written as:

(8) $$\chi_{\left(i\right)}^{\mathrm{mech}}\propto \sum_{n}\frac{\partial^{3}V}{\partial Q_{a}\partial Q_{n}\partial Q_{b}}\left[Tr\left(\frac{\partial \alpha }{\partial Q_{b}}\right)\left(\frac{\partial \mu }{\partial Q_{n}}.\frac{\partial \mu }{\partial Q_{a}}\right)+2\frac{\partial \alpha }{\partial Q_{b}}.\frac{\partial \mu }{\partial Q_{n}}.\frac{\partial \mu }{\partial Q_{a}}\right]$$

(9) $$\chi_{\left(i\right)}^{elect}\propto Tr\left(\frac{\partial \alpha }{\partial Q_{b}}\right)\left(\frac{\partial^{2}\mu }{\partial Q_{a}\partial Q_{b}}.\frac{\partial \mu }{\partial Q_{a}}\right)+2\left(\frac{\partial \alpha }{\partial Q_{b}}.\frac{\partial^{2}\mu }{\partial Q_{a}\partial Q_{b}}\right).\frac{\partial \mu }{\partial Q_{a}}.$$

The output signal intensity is proportional to the absolute square of the third-order non-linear susceptibility from different states a and b:

(10) $$S\left(\omega_{1},\omega_{2}\right)\propto {\left|\sum_{a,b}\chi_{\left(i\right)}^{\left(3\right)}\left(\omega_{1},\omega_{2}\right)\right|}^{2}.$$

### 4.2. DFT calculations and EVV-2DIR Spectroscopy Theoretical Simulation

The geometry optimizations and frequency calculations were carried out use hybrid density functional theory (DFT) at the level of M062X [41] with the 6-31G (d) basis sets [42] in the Gaussian 09 program (Revision D.01; Gaussian, Inc: Wallingford, CT, 2013) [43]. The SMD model with toluene as a solvent was used to consider the solvation effects [44]. The frequency scale factor 0.947 was used to correct the harmonic frequencies throughout the IR, Raman and EVV-2DIR spectrum. Only the coherence pathway (i) was considered herein. We applied the same theoretical method as used by Kwak. et al [45] to calculate the EVV-2DIR signal intensity χ^(3)^ and didn’t consider the contributions from “promoting modes” since their contributions are negligible. The two IR frequencies step size is 5 cm^−1^. Each cross peak in EVV-2DIR spectrum was fitted with a 2D Gaussian function with harmonic frequency as the center frequency and the half-width at half height (HWHM) of 10 cm^−1^. 

## Figures and Tables

**Table 1:** The vibrational mode pairs corresponding to the characteristic cross peaks in the β-aminoxylated LM2A and their respective contributions.

Peak Label	Coupling Modes/cm^−1^	Vibrational Assignment^1^	χ^mech^	χ^elect^	χ^(3)^
A	1050.2/2030.7	asymm CO stretch/ asymm CO stretch + CH_2_ wag (81%)	1.51 × 10^−15^	−2.28 × 10^−16^	1.28 × 10^−15^
	1050.2/2047.8	asymm CO stretch/ asymm CO stretch + CH_3_ wag (17%)	6.70 × 10^−16^	−2.07 × 10^−16^	4.63 × 10^−16^
B	1050.2/2700.3	asymm CO stretch/ asymm CO stretch + C=C stretch (97%)	7.31 × 10^−17^	−2.83 × 10^−15^	−2.76 × 10^−15^
C	1334.0/2984.1	CH rock/CH rock + C=C stretch (32%)	−1.56 × 10^−16^	−7.42 × 10^−16^	−8.97 × 10^−16^
	1332.6/2982.7	CH rock/CH rock + C=C stretch (28%)	−3.86 × 10^−17^	−7.81 × 10^−16^	−8.20 × 10^−16^
	1326.6/2976.7	CH_2_ wag/CH_2_ wag + C=C stretch (26%)	−1.12 × 10^−16^	−6.58 × 10^−16^	−7.70 × 10^−16^
D	1650.1/3300.2	C=C stretch/C=C stretch + C=C stretch (100%)	5.31 × 10^−15^	−1.60 × 10^−15^	3.71 × 10^−15^

^1^ Numbers in parentheses are the contribution ratio of vibration mode pair to the corresponding peak.

**Table 2:** The vibrational mode pairs corresponding to the characteristic cross peaks in the enamine tautomer LM1B and their respective contributions.

Peak Label	Coupling Modes/cm^−1^	Vibrational Assignment ^1^	χ^mech^	χ^elect^	χ^(3)^
E	1192.6/2385.2	NH rock/NH rock + NH rock (100%)	−9.59 × 10^−16^	−2.08 × 10^−15^	−3.04 × 10^−15^
F	1192.6/2870.4	NH rock/NH rock + C=C stretch	−5.14 × 10^−18^	−4.96 × 10^−15^	−4.97 ×10^−15^
G	1365.5/3043.3	CH_2_ wag/CH_2_ wag + C=C stretch (100%)	−7.04 × 10^−16^	−1.55 × 10^−15^	−2.26 × 10^−15^
H	1677.8/2870.4	C=C stretch/C=C stretch + NH rock (100%)	1.45 × 10^−17^	−1.86 × 10^−15^	−1.85 × 10^−15^
I	1677.8/4556.6	C=C stretch/C=C stretch + CH_2_ symm stretch (96%)	1.65 × 10^−16^	−2.02 × 10^−15^	−1.86 × 10^−15^

^1^ Numbers in parentheses are the contribution ratio of vibration mode pair to the corresponding peak.

**Table 3:** The vibrational mode pairs corresponding to the characteristic cross peaks in the α-aminoxylated intermediate LM3B and their respective contributions.

Peak Label	Coupling Modes /cm^−1^	Vibrational Assignment ^1^	χ^mech^	χ^elect^	χ^(3)^
J	1074.4/4037.2	C=N stretch/ C=N stretch + CH_2_ asymm stretch (31%)	4.08 × 10^−17^	−3.19 × 10^−16^	−2.79 × 10^−16^
	1074.4/4026.2	C=N stretch/ C=N stretch + CH_2_ asymm stretch (24%)	−2.06 × 10^−17^	−2.12 × 10^−16^	−2.33 × 10^−16^
	1074.4/4022.4	C=N stretch/ C=N stretch + CH_2_ asymm stretch (21%)	3.35 × 10^−17^	−2.51 × 10^−16^	−2.17 × 10^−16^
	1091.8/4043.6	CH_2_ twist/ CH_2_ twist + CH_2_ asymm stretch (16%)	−1.99 × 10^−18^	−1.77 × 10^−16^	−1.79 × 10^−16^
	1091.8/4054.5	CH_2_ twist/ CH_2_ twist + CH_2_ asymm stretch (9%)	−6.30 × 10^−17^	−5.93 × 10^−17^	−1.22 × 10^−16^
K	1723.5/2954.3	C=N stretch/ C=N stretch + CH_2_ twist (100%)	2.33 × 10^−16^	9.03 × 10^−16^	1.14 × 10^−15^
L	1723.5/3446.9	C=N stretch/ C=N stretch + C=N stretch (100%)	4.71 × 10^−15^	4.63 × 10^−15^	9.34 × 10^−15^

^1^ Numbers in parentheses are the contribution ratio of vibration mode pair to the corresponding peak.

**Table 4:** The vibrational mode pairs corresponding to the characteristic cross peaks in the α-aminoxylated intermediate LM4B and their respective contributions.

Peak Label	Coupling Modes/cm^−1^	Vibrational Assignment ^1^	χ^mech^	χ^elect^	χ^(3)^
M	1124.5/2820.0	CH_2_ wag/ CH_2_ wag + C=C stretch (94%)	−3.87 × 10^−16^	−3.89 × 10^−15^	−4.28 × 10^−15^
N	1124.5/4427.1	CH_2_ wag/ CH_2_ wag + NH stretch (96%)	−1.11× 10^−16^	2.36 × 10^−15^	2.25 × 10^−15^
O	1203.0/2898.5	CH_2_ twist/ CH_2_ twist + C=C stretch (99%)	−2.79 × 10^−16^	−1.90 × 10^−15^	−2.18 × 10^−15^
P	1342.5/3038.1	CH_2_ wag/ CH_2_ wag + C=C stretch (74%)	−1.29 × 10^−16^	−1.25 × 10^−15^	−1.38 × 10^−15^
	1349.7/3045.3	CH_2_ wag/ CH_2_ wag + C=C stretch (25%)	−3.78 × 10^−17^	−6.12 × 10^−16^	−6.50 × 10^−16^
Q	1414.3/4717.0	CH_2_ scissor/CH_2_ scissor + NH stretch (47%)	2.97 × 10^−16^	−3.47 × 10^−15^	−3.18 × 10^−15^
	1426.4/4729.0	CH_2_ scissor/CH_2_ scissor +NH stretch (23%)	2.27 × 10^−16^	−2.10 × 10^−15^	−1.88 × 10^−15^
	1419.3/4721.9	CH_2_ scissor/CH_2_ scissor +NH stretch (22%)	2.64 × 10^−16^	−2.10 × 10^−15^	−1.83 × 10^−15^

^1^ Numbers in parentheses are the contribution ratio of vibration mode pair to the corresponding peak.

## Supplementary Materials

The following are available online, Figure S1: Calculated IR and Raman spectrum for each species in enamine reaction pathway. Figure S2: Calculated IR and Raman spectrum for each species in imine reaction pathway.
