# Supplementary material for: Differentiation between Enamines and Tautomerizable Imines Oxidation Reaction Mechanism using Electron-Vibration-Vibration Two Dimensional Infrared Spectroscopy
Source: Molecules. 2019 Mar 1;24(5):869. doi: 10.3390/molecules24050869 (PMC6429144; doi:10.3390/molecules24050869)
Supplement: Supplementary file 1 [file molecules-24-00869-s001.pdf]

Supporting Information for:

Differentiation between enamines and tautomerizable imines oxidation reaction mechanism using Electron-vibration-vibration two dimensional infrared spectroscopy

Fengqin Long [1,2], Zheng Chen [3,4], Keli Han [1,], Lu Zhang [3,*] and Wei Zhuang [3,*]*

[1] State Key Laboratory of Molecular Reaction Dynamics, Dalian Institute of Chemical Physics, Chinese Academy of Science, Dalian, Liaoning 116023, China; fqlong@dicp.ac.cn

[2] University of the Chinese Academy of Sciences, Beijing 100049, China

[3] State Key Laboratory of Structural Chemistry, Fujian Institute of Research on the Structure of Matter, Chinese Academy of Sciences, Fuzhou, Fujian 350002, China; jeack1808@126.com

[4] Collaborative Innovation Center of Chemistry for Energy Materials, Shanghai Key Laboratory of Molecular Catalysis and Innovative Materials, MOE Key Laboratory of Computational Physical Sciences, Department of Chemistry, Fudan University, Shanghai 200433, China

---

* To whom correspondence should be addressed: klhan@dicp.ac.cn; luzhang@fjirsm.ac.cn; wzhuang@fjirsm.ac.cn

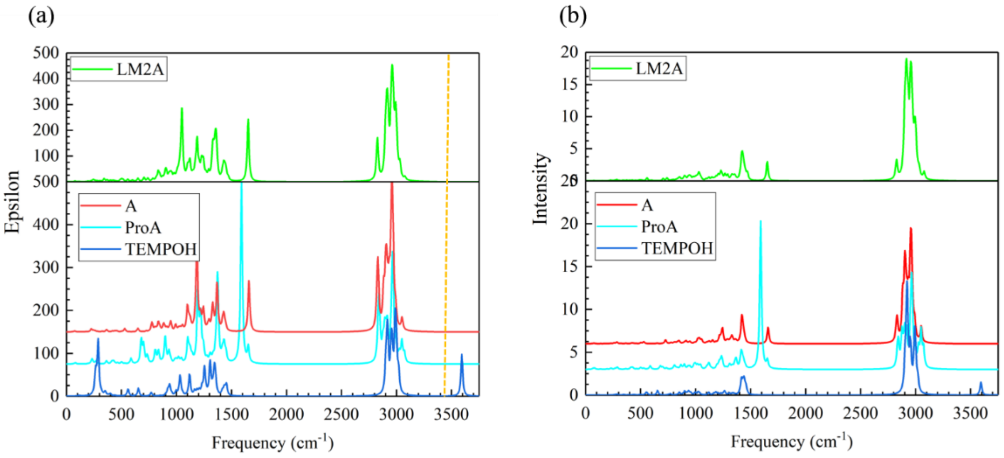

Figure S1. Calculated (a) IR and (b) Raman spectrum for each species in enamine reaction pathway. The half-width at half height of IR and Raman peaks is 10 cm^{-1}.

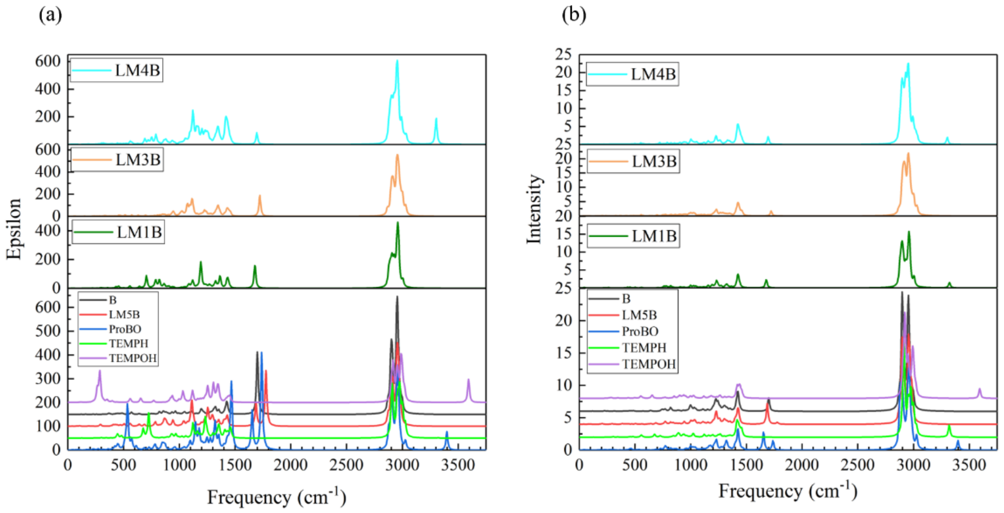

Figure S2. Calculated IR (a) and Raman (b) spectrum for each species in imine reaction pathway. The half-width at half height of IR and Raman peaks is 10 cm^{-1}.
